# Acute and Chronic Mood and Apathy Outcomes from a Randomized Study of Unilateral STN and GPi DBS

**DOI:** 10.1371/journal.pone.0114140

**Published:** 2014-12-03

**Authors:** Michael S. Okun, Samuel S. Wu, Sarah Fayad, Herbert Ward, Dawn Bowers, Christian Rosado, Lauren Bowen, Charles Jacobson, Christopher Butson, Kelly D. Foote

**Affiliations:** 1 Department of Neurology, Center for Movement Disorders and Neurorestoration, University of Florida, Gainesville, Gainesville, FL, United States of America; 2 Department of Neurosurgery, Center for Movement Disorders and Neurorestoration, University of Florida, Gainesville, Gainesville, FL, United States of America; 3 Department of Biostatistics, Center for Movement Disorders and Neurorestoration, University of Florida, Gainesville, Gainesville, FL, United States of America; 4 Department of Psychiatry, Center for Movement Disorders and Neurorestoration, University of Florida, Gainesville, Gainesville, FL, United States of America; 5 Department of Clinical and Health Psychology, Center for Movement Disorders and Neurorestoration, University of Florida, Gainesville, Gainesville, FL, United States of America; 6 Department of Neurology, Medical College of Wisconsin, Milwaukee, WI, United States of America; INSERM / CNRS, France

## Abstract

**Objective:**

To study mood and behavioral effects of unilateral and staged bilateral subthalamic nucleus (STN) and globus pallidus internus (GPi) deep brain stimulation (DBS) for Parkinson's disease (PD).

**Background:**

There are numerous reports of mood changes following DBS, however, most have focused on bilateral simultaneous STN implants with rapid and aggressive post-operative medication reduction.

**Methods:**

A standardized evaluation was applied to a subset of patients undergoing STN and GPi DBS and who were also enrolled in the NIH COMPARE study. The Unified Parkinson Disease Rating Scale (UPDRS III), the Hamilton depression (HAM-D) and anxiety rating scales (HAM-A), the Yale-Brown obsessive-compulsive rating scale (YBOCS), the Apathy Scale (AS), and the Young mania rating scale (YMRS) were used. The scales were repeated at acute and chronic intervals. A post-operative strategy of non-aggressive medication reduction was employed.

**Results:**

Thirty patients were randomized and underwent unilateral DBS (16 STN, 14 GPi). There were no baseline differences. The GPi group had a higher mean dopaminergic dosage at 1-year, however the between group difference in changes from baseline to 1-year was not significant. There were no differences between groups in mood and motor outcomes. When combining STN and GPi groups, the HAM-A scores worsened at 2-months, 4-months, 6-months and 1-year when compared with baseline; the HAM-D and YMRS scores worsened at 4-months, 6-months and 1-year; and the UPDRS Motor scores improved at 4-months and 1-year. Psychiatric diagnoses (DSM-IV) did not change. No between group differences were observed in the cohort of bilateral cases.

**Conclusions:**

There were few changes in mood and behavior with STN or GPi DBS. The approach of staging STN or GPi DBS without aggressive medication reduction could be a viable option for managing PD surgical candidates. A study of bilateral DBS and of medication reduction will be required to better understand risks and benefits of a bilateral approach.

## Introduction

Deep brain stimulation (DBS) is the most frequently performed surgical intervention for appropriately screened advanced idiopathic Parkinson's disease (PD) patients [1]. The subthalamic nucleus (STN) and globus pallidus internus (GPi) DBS are effective surgical targets, and both provide superior motor outcomes when compared to best medical management in carefully selected patients with motor fluctuations [2] [3]–[5]. To date, the most widely adopted surgical approach has been bilateral simultaneous STN electrode implantation [6]. Recently, data has emerged suggesting that single lead (unilateral) implantation may have an expanding role in the treatment of advanced PD, and that many DBS candidates have an excellent outcome with a single DBS lead [5], [7].

Growing evidence has revealed that PD patients receiving either unilateral or bilateral STN DBS will possibly experience post-operative DBS-related mood changes [3]–[5], [8]–[10]. Additionally, several recent studies have suggested that rapid and aggressive post-operative medication reduction following bilateral STN DBS may result in apathy, anxiety, depression, and other behavioral issues [11], [12]. In the current study we sought to investigate beyond the original NIH COMPARE study, both the acute and chronic mood issues in patients implanted with unilateral STN or GPi DBS. Additionally, we also further documented the findings in cases proceeding to staged bilateral STN or GPi DBS placement. We employed a battery of validated mood and behavioral instruments collected at baseline, as well as several pre-determined acute and chronic time points following DBS placement in order to better understand the time course of mood issues in both the STN and GPi targets. The original NIH COMPARE study [13] did not report acute and chronic mood changes in detail.

## Methods

A standardized study protocol for patient screening, subject enrollment, data collection and data analysis was implemented. This observational study was registered at clinical trials.gov NCT00954772, and this was an IRB approved study by the University of Florida IRB and patients provided consent for their information to be published. Patient records were anonymized and de-identified prior to analysis. Study subjects were recruited from the UF Center for Movement Disorders and Neurorestoration clinics. Every study subject was scheduled to receive DBS therapy based on standard clinical criteria, all subjects had to be ≤75 years of age, and all subjects were evaluated by a complete interdisciplinary team (neurologist, neurosurgeon, neuropsychologist, psychiatrist, physical therapist, occupational therapist, speech therapist) and the DBS target was randomized between STN and GPi. All subjects signed an Institutional Review Board (IRB) approved informed consent form and the study was an IRB approved study. No reimbursements or other benefits were offered. Subjects retained the right to refuse participation in the protocol after the DBS surgical procedure. Patients also retained the option for implantation of the contralateral side after a minimum of 6-months of stimulation contingent on if they met the standard criteria for DBS eligibility and had symptom(s) potentially addressable by a contralateral DBS lead.

DBS leads were inserted into the STN or GPi targets using standard neurosurgical technique. Subjects remained awake during the procedure. Targeting was performed pre-operatively using CT and MRI fusion. Intra-operative microelectrode guidance and target-confirmation was performed using multi-pass, microelectrode recording and brain-mapping. An experienced functional neurosurgeon and a DBS-trained movement disorders specialist performed every procedure (KDF and MSO). Subjects initially had a single DBS lead implanted to address either the most bothersome side of the body or alternatively to address the dominant hand.

Prior to surgery, the general characteristics of the subjects were collected, including demographic data and the Unified Parkinson Disease Rating Scale (UPDRS) scores. In addition, several validated mood and behavioral instruments were collected at baseline (within one-month prior to surgery), 2-weeks, 4-weeks, 2-months, 4-months, and 6-months following each DBS placement (repeated after the second lead if placed contralaterally). Assessments included the Hamilton depression rating scale (HAM-D), the Hamilton anxiety rating scale (HAM-A), the Yale-Brown obsessive-compulsive scale (YBOCS), the Young mania rating scale (YMRS), the Apathy Scale (AS), the Beck Depression Inventory-II (BDI-II), and the Beck Anxiety Inventory (BAI) [14]–[19]. The number of subjects who missed baseline mood measures ranged from 1 for BDI to 11 for YBOCS. Patients were managed post-operatively at monthly intervals. Patients were counseled that medications would be used in combination with DBS to maximize benefits and to minimize side effects. The medication reduction strategy included increasing intervals between medication dosages, discontinuing entacapone, discontinuing amantadine, and decreasing medication dosages (levodopa and/or agonists). Medication reduction strategies were deliberately employed slowly, and all changes monitored by clinicians monthly. Rapid and aggressive medication discontinuation strategies were not employed unless severe and difficult to manage dyskinesia was encountered. The study was started after the COMPARE study had been initiated and thus includes a smaller number of identical subjects.

### Statistical Analysis

Following the study completion, data were analyzed qualitatively and quantitatively with SAS software. The mood and cognitive outcomes were compared between groups (unilateral vs. bilateral) using Wilcoxon rank sum test at each follow-up; while within-group changes from baseline were assessed using the signed rank test. In addition, general linear models were fitted with changes of levodopa equivalent dose (LED) and mood or motor outcomes from baseline to 1-year follow-up as dependent variables, and DSM psychiatric diagnosis of independent variable, controlling for age, gender, years with PD symptoms, lateral and target of stimulation. Missing values were imputed based on baseline characteristics and outcomes observed ahead of them.

## Results

### A. Results of Unilateral STN and GPi DBS Cases

There were 30 patients who underwent unilateral DBS (16 STN, 14 GPi), and there were no baseline demographic, disease, or other differences between groups (see Table 1).

**Table 1:** Baseline Characteristics.

variable		All (N = 30)	GPi (N = 14)	STN (N = 16)	P-value
Age		59.0±8.6 (N = 30)	60.1±5.5 (N = 14)	58.0±10.7 (N = 16)	0.519
Gender	F	9 (30.0%)	6 (42.9%)	3 (18.8%)	0.151
	M	21 (70.0%)	8 (57.1%)	13 (81.3%)	
Years with Symptom		11.8±3.9 (N = 30)	11.5±3.3 (N = 14)	12.1±4.5 (N = 16)	0.669
Baseline off med HY stage	2	7 (25.9%)	5 (41.7%)	2 (13.3%)	0.222
	2.5	7 (25.9%)	2 (16.7%)	5 (33.3%)	
	3	10 (37.0%)	4 (33.3%)	6 (40.0%)	
	4	2 (7.4%)		2 (13.3%)	
	5	1 (3.7%)	1 (8.3%)		
Baseline on med HY stage	1	1 (3.6%)	1 (7.1%)		0.498
	2	23 (82.1%)	11 (78.6%)	12 (85.7%)	
	2.5	3 (10.7%)	1 (7.1%)	2 (14.3%)	
	3	1 (3.6%)	1 (7.1%)		
DSM Diagnosis	Yes	27 (90.0%)	13 (92.9%)	14 (87.5%)	0.626
	No	3 (10.0%)	1 (7.1%)	2 (12.5%)	
	Anxiety	11 (36.7%)	4 (28.6%)	7 (43.8%)	0.466
	Apathy	8 (26.7%)	2 (14.3%)	6 (37.5%)	0.226
	Depression	15 (50.0%)	6 (42.9%)	9 (56.3%)	0.715
LED		917.2±550.6 (N = 29)	1037.1±647.8 (N = 14)	805.4±434.7 (N = 15)	0.265
Off med Motor score		42.7±12.7 (N = 30)	41.4±10.3 (N = 14)	43.9±14.8 (N = 16)	0.598
On med Motor score		21.5±8.9 (N = 30)	20.8±8.3 (N = 14)	22.1±9.6 (N = 16)	0.688
Off-on Motor score change		−21.2±8.8 (N = 30)	−20.6±5.6 (N = 14)	−21.8±11.0 (N = 16)	0.721
HAM-A		2.2±3.8 (N = 21)	2.0±2.9 (N = 11)	2.5±4.8 (N = 10)	0.774
HAM-D		1.5±1.9 (N = 22)	1.3±2.0 (N = 12)	1.6±1.9 (N = 10)	0.754
BDI		6.6±4.7 (N = 29)	6.1±3.6 (N = 14)	7.1±5.6 (N = 15)	0.551
AS		10.1±6.0 (N = 27)	9.8±6.2 (N = 12)	10.3±6.1 (N = 15)	0.857
YBOCS		0.0±0.0 (N = 19)	0.0±0.0 (N = 9)	0.0±0.0 (N = 10)	1.000
YMRS		0.9±2.5 (N = 22)	0.3±0.6 (N = 12)	1.6±3.7 (N = 10)	0.221

LED- levodopa equivalent dose; HAM-A- Hamilton Anxiety Inventory; HAM-D- Hamilton Depression Inventory; BDI- Beck Depression Inventory; AES- Apathy Scale; YBOCS- Yale Brown Obsessive Compulsive Scale; YMRS- Young Mania Rating Scale; UPDRS- Unified Parkinson's Disease Rating Scale Motor Section (III); H and Y- Hoehn and Yahr Parkinson Stage; DSM- Diagnostic and Statistical Manual Psychiatric Mood Diagnosis; Time intervals correspond to time from the DBS operation.

### Comparison of Outcomes of Unilateral STN versus Unilateral GPi DBS

Table 2 presents the median, mean and standard deviation (SD) of outcomes by group over time. The GPi group had a higher LED than the STN group at 1-year, however the between group difference in change from baseline to 1-year was not significant because the GPi group started with a higher LED. No significant difference was found between the two groups in mood and motor outcomes.

**Table 2:** Comparison of Outcomes in Unilateral STN versus Unilateral GPi DBS.

Variable	Type	Baseline (Median, Mean ± SD)	2-month		4-month		6-month		1-year	
			Median, Mean ± SD	Change	Median, Mean ± SD	Change	Median, Mean ± SD	Change	Median, Mean ± SD	Change
LED	STN	801, 820±456			934, 1008±464	17.0, 120±360			800, 802±433	110, −17.9±599
	GPi	1000, 1150±692			1157, 1217±517	150, 66.5±361			1600, 1580±763	250, 430±642*
	p-value	0.323			0.438	0.970			**0.020**	0.183
HAM-A	STN	2.00, 4.00±5.16	3.50, 6.50±5.48	1.00, 2.50±5.23	8.00, 9.80±8.02	3.50, 5.80±8.24*	6.50, 10.9±12.1	3.50, 6.90±12.6	6.50, 11.1±11.9	3.50, 7.10±12.5*
	GPi	0, 2.09±2.95	4.50, 5.50±6.00	3.50, 4.00±4.90*	4.00, 6.20±7.04	3.50, 4.70±5.33*	4.50, 6.70±7.13	4.50, 5.20±5.37*	7.00, 8.30±6.70	6.00, 6.80±5.32*
	p-value	0.478	0.819	0.210	0.210	1.000	0.516	0.733	0.940	0.405
HAM-D	STN	1.00, 2.20±2.62	2.50, 2.40±2.01	0, 0.20±2.94	4.50, 5.00±3.89	1.50, 2.80±4.42	4.50, 5.90±6.44	1.50, 3.70±6.52*	4.50, 5.80±6.43	1.50, 3.60±6.57
	GPi	1.00, 2.18±3.06	1.00, 3.60±6.00	0, 1.30±3.71	2.00, 3.20±4.42	1.00, 0.90±2.96	2.50, 2.90±3.35	1.00, 0.60±2.59	3.00, 3.30±3.16	2.00, 1.00±2.62
	p-value	0.941	0.788	0.905	0.170	0.380	0.340	0.285	0.540	0.540
BDI	STN	4.50, 7.10±6.52			5.00, 8.44±6.89	−1.00, 0.78±3.96			6.00, 8.56±7.94	0, 0.89±6.53
	GPi	7.00, 7.27±2.94			3.00, 6.91±6.09	−1.00, −0.36±5.95			4.00, 6.09±6.22	−1.00, −1.18±5.93
	p-value	0.376			0.592	0.540			0.379	0.267
AS	STN	10.0, 10.7±6.13			17.0, 16.4±9.28	6.00, 5.78±7.50			17.0, 13.9±7.85	5.00, 3.22±8.11
	GPi	9.00, 10.5±6.33			11.0, 13.1±5.99	3.00, 3.00±5.08			11.0, 13.0±5.89	2.50, 2.50±4.12
	p-value	0.879			0.422	0.487			0.622	0.806
YBOCS	STN	0, 0±0			0, 1.50±3.81	0, 1.50±3.81	0, 2.60±7.23	0, 2.60±7.23	0, 2.60±7.23	0, 2.60±7.23
	GPi	0, 0±0			0, 0.90±1.91	0, 0.90±1.91	0, 0.50±1.58	0, 0.50±1.58	0, 0.50±1.58	0, 0.50±1.58
	p-value	1.000			1.000	1.000	0.584	0.584	0.584	0.584
YMRS	STN	0, 1.40±3.10	0.50, 1.70±2.16	0, 0.30±2.00	1.00, 2.00±2.58	1.00, 0.60±2.55	1.00, 2.40±3.47	1.00, 1.00±2.45	1.00, 2.50±3.44	1.00, 1.10±2.47
	GPi	0, 0.36±0.67	1.00, 1.60±1.84	1.00, 1.40±1.84*	1.00, 2.00±2.00	1.00, 1.80±1.99*	1.00, 2.30±2.50	1.00, 2.10±2.51*	1.00, 2.30±2.50	1.00, 2.10±2.56*
	p-value	0.962	0.783	0.194	1.000	0.487	1.000	0.727	0.907	0.700
UPDRS Motor-Off	STN	41.5, 41.2±9.32			30.0, 31.2±9.75	−9.50, −10.0±5.66*			29.5, 29.7±10.4	−10.5, −11.5±5.50*
	GPi	38.0, 40.5±11.2			33.0, 33.9±9.89	−9.00, −6.55±8.05*			33.0, 33.9±12.1	−6.00, −6.55±6.58*
	p-value	0.437			0.672	0.438			0.397	0.104
UPDRS-Motor On	STN	20.0, 21.3±7.56			22.0, 22.3±9.64	2.50, 1.00±6.57			20.5, 22.6±8.21	3.00, 1.30±6.88
	GPi	20.0, 20.8±8.68			23.0, 22.1±7.69	1.00, 1.27±6.28			23.0, 22.7±6.56	1.00, 1.91±4.70
	p-value	0.672			1.000	0.860			0.860	0.944

LED- levodopa equivalent dose; HAM-A- Hamilton Anxiety Inventory; HAM-D- Hamilton Depression Inventory; BDI- Beck Depression Inventory; AES- Apathy Scale; YBOCS- Yale Brown Obsessive Compulsive Scale; YMRS- Young Mania Rating Scale; UPDRS- Unified Parkinson's Disease Rating Scale Motor Section (III); Time intervals correspond to time from the DBS operation. Within group changes were tested using the signed rank test and significant results are highlighted in bold (* for p<0.05; and ** for p<0.01); while the between group comparisons were tested using the Wilcoxon rank sum test and corresponding p-values presented in the 3^rd^ row of each block.

### Comparison of Outcomes Over Time for All Unilateral DBS Cases

Since the STN and GPi groups did not have significant differences, we compared outcomes overtime for all unilateral cases. Table 3 revealed that: HAM-A scores significantly increased at 2-months, 4-months, 6-months and 1-year when compared with baseline; HAM-D and YMRS scores significantly increased at 4-months, 6-months and 1-year; and UPDRS Motor scores significantly decreased (improved) at 4-months and 1-year when compared with baseline (comparing baseline off meds to the off meds on DBS conditions).

**Table 3:** Comparison of Outcomes Over Time for All Unilateral DBS Cases (Including both STN and GPi).

Variable	Baseline	2-month			4-month			6-month			1-year		
		Median, Mean ± SD	Change	p-value	Median, Mean ± SD	Change	p-value	Median, Mean ± SD	Change	p-value	Median, Mean ± SD	Change	p-value
LED	868, 1002±607				1100, 1117±492	96.0, 90.4±352	0.242				1150, 1230±737	208, 228±648	0.068
HAM-A	2.00, 3.00±4.16	4.00, 6.00±5.62	**1.50, 3.25±4.99**	**0.006**	5.00, 8.00±7.57	**3.50, 5.25±6.78**	**0.0003**	5.00, 8.80±9.90	**4.00, 6.05±9.47**	**0.0009**	6.50, 9.70±9.51	**5.00, 6.95±9.35**	**0.0003**
HAM-D	1.00, 2.19±2.79	2.00, 3.00±4.40	0, 0.75±3.31	0.403	2.50, 4.10±4.15	**1.00, 1.85±3.79**	**0.028**	3.00, 4.40±5.23	**1.50, 2.15±5.08**	**0.027**	3.00, 4.55±5.09	**2.00, 2.30±5.05**	**0.017**
BDI	6.00, 7.19±4.84				4.50, 7.60±6.34	−1.00, 0.15±5.06	0.960				4.00, 7.20±6.96	−1.00, −0.25±6.13	0.515
AS	10.0, 10.6±6.06				15.0, 14.6±7.62	5.00, 4.32±6.32	0.008				14.0, 13.4±6.70	3.00, 2.84±6.15	0.066
YBOCS	0, 0±0	0, 0.55±2.04	0, 0.55±2.04	0.500	0, 1.20±2.95	0, 1.20±2.95	0.125	0, 1.55±5.21	0, 1.55±5.21	0.250	0, 1.55±5.21	0, 1.55±5.21	0.250
YMRS	0, 0.86±2.20	1.00, 1.65±1.95	0, 0.85±1.95	0.051	1.00, 2.00±2.25	**1.00, 1.20±2.31**	**0.020**	1.00, 2.35±2.94	**1.00, 1.55±2.48**	**0.006**	1.00, 2.40±2.93	**1.00, 1.60±2.50**	**0.005**
UPDRS Motor-Off	39.0, 40.8±10.1				33.0, 32.6±9.68	**−9.00, −8.19±7.07**	**<.0001**				32.0, 31.9±11.3	**−9.00, −8.90±6.46**	**<.0001**
UPDRS-Motor On	20.0, 21.0±7.97				23.0, 22.2±8.45	2.00, 1.14±6.26	0.468				23.0, 22.7±7.20	2.00, 1.62±5.70	0.205

LED- levodopa equivalent dose; HAM-A- Hamilton Anxiety Inventory; HAM-D- Hamilton Depression Inventory; BDI- Beck Depression Inventory; AS- Apathy Scale; YBOCS- Yale Brown Obsessive Compulsive Scale; YMRS- Young Mania Rating Scale; UPDRS- Unified Parkinson's Disease Rating Scale Motor Section (III); Time intervals correspond to time from the DBS operation. Significant changes are highlighted in bold (p<0.05; and for p<0.01).

### Pre-operative DSM Diagnoses

Psychiatric Diagnoses according to DSM-IV criteria were not associated with changes of any mood or motor scale. Female patients had a larger increase in HAM-A (9.3 points more, p = 0.008), HAM-D (5.5 points more, p = 0.004), and YBOCS (4.0 points more, p = 0.016) at one-year follow-up. In addition, years with PD symptoms (p = 0.017) and age at 1^st^ surgery (p = 0.0015) were negatively associated with changes in YBOCS.

### Correlation Between LED Change From Baseline to One Year and Mood/Behavioral Changes

There was a significant positive correlation between changes in LED and HAM-A (Spearman's rank correlation r = 0.518, p = 0.007). However, the LED change was not correlated with other mood and motor scores.

### B. Results of Bilateral STN and Bilateral GPi DBS Cases

Of the STN group, 6 (37.5%) proceeded to bilateral DBS, and 3 (21.4%) of the GPi cohort received a second lead following 6 months of stimulation. Similar to unilateral cases, a comparison of bilateral GPi and STN has been summarized in Table 4. LED was not statistically significantly decreased in either target in bilaterally implanted patients. No significant between group differences were found, therefore we compared outcomes overtime for all bilateral cases. Significant improvements in UPDRS scores were observed following 4 months and one year of stimulation (see Tables 5). Due to the small number of bilateral cases, we did not perform regression analysis on mood changes or correlation analysis between mood changes and LED. Table 6 summarizes the lack of correlation with LED change.

**Table 4:** Comparison of Outcomes of Bilateral STN versus Bilateral GPi DBS.

Variable	Type	Baseline (Median, Mean ± SD)	2-month		4-month		6-month		1-year	
			Median, Mean ± SD	Change	Median, Mean ± SD	Change	Median, Mean ± SD	Change	Median, Mean ± SD	Change
LED	STN	950, 784±441			844, 781±281	−0.01, −2.34±284			550, 775±546	199, −8.59±637
	GPi	600, 622±69.4			517, 517±23.6	−117, −117±70.7			467, 467±47.1	−167, −167±141
	p-value	0.519			0.500	0.405			0.393	0.617
HAM-A	STN	2.00, 4.17±5.88	6.00, 9.40±10.0	3.00, 4.40±6.39	8.00, 11.0±9.30	6.00, 6.00±6.96	9.00, 12.6±10.8	6.00, 7.60±8.59	7.00, 10.0±10.5	5.00, 5.00±5.83
	GPi	2.00, 2.00±2.00	1.00, 4.33±6.66	1.00, 2.33±5.13	1.00, 3.33±4.93	1.00, 1.33±3.51	0, 2.33±4.04	0, 0.33±2.52	0, 2.33±4.04	0, 0.33±2.52
	p-value	0.892	0.453	0.764	0.294	0.371	0.134	0.294	0.175	0.294
HAM-D	STN	2.50, 2.50±1.52	2.00, 6.00±6.48	0, 3.00±6.56	3.00, 6.00±6.48	1.00, 3.00±6.89	4.00, 6.40±5.22	2.00, 3.40±5.50	6.00, 6.20±3.49	3.00, 3.20±3.56
	GPi	0, 0.33±0.58	1.00, 0.67±0.58	0, 0.33±0.58	2.00, 2.00±2.00	1.00, 1.67±2.08	2.00, 1.33±1.15	1.00, 1.00±1.00	2.00, 1.33±1.15	1.00, 1.00±1.00
	p-value	0.085	0.172	1.000	0.549	1.000	0.112	0.881	0.067	0.294
BDI	STN	6.50, 6.00±4.38			3.00, 3.17±2.79	−3.00, −2.83±5.56			5.50, 6.17±5.12	−0.50, 0.17±5.23
	GPi	1.00, 1.67±2.08			2.00, 2.00±2.00	0, 0.33±1.53			0, 1.67±2.89	0, 0±1.00
	p-value	0.193			0.695	0.298			0.154	1.000
AS	STN	7.00, 8.83±6.08			10.0, 11.8±6.57	2.00, 3.40±6.47			19.0, 18.2±9.07	6.00, 9.80±9.18
	GPi	8.00, 6.67±4.16			4.00, 5.67±5.69	2.00, −1.00±7.00			4.00, 6.00±5.29	2.00, −0.67±6.43
	p-value	0.795			0.230	0.653			0.134	0.134
YMRS	STN	0, 2.33±4.41	6.00, 5.80±3.96	1.00, 3.00±3.74	3.00, 3.20±0.45	3.00, 0.40±4.93	3.00, 3.20±2.28	1.00, 0.40±2.70	2.00, 1.80±1.30	0, −1.00±4.64
	GPi	0, 0±0	0, 1.00±1.73	0, 1.00±1.73	1.00, 1.33±1.53	1.00, 1.33±1.53	0, 1.00±1.73	0, 1.00±1.73	0, 1.00±1.73	0, 1.00±1.73
	p-value	0.377	0.097	0.525	0.088	0.878	0.219	1.000	0.531	0.873
UPDRS Motor-Off	STN	45.0, 48.3±21.5			29.5, 30.3±8.29	−12.5, −18.0±15.5*			22.0, 26.8±10.9	−15.5, −21.5±15.3*
	GPi	45.0, 44.7±5.51			30.0, 33.3±12.3	−9.00, −11.3±9.71			30.0, 30.3±0.58	−15.0, −14.3±5.03
	p-value	1.000			0.795	0.519			0.515	0.897
UPDRS-Motor On	STN	20.0, 23.5±13.1			19.0, 22.0±10.7	0, −1.50±14.0			16.5, 21.2±8.89	1.00, −2.33±6.77
	GPi	17.0, 20.7±8.14			26.0, 28.0±4.36	9.00, 7.33±3.79			27.0, 29.0±4.36	9.00, 8.33±11.0
	p-value	1.000			0.362	0.245			0.300	0.243

LED- levodopa equivalent dose; HAM-A- Hamilton Anxiety Inventory; HAM-D- Hamilton Depression Inventory; BDI- Beck Depression Inventory; AS- Apathy Scale; YBOCS- Yale Brown Obsessive Compulsive Scale; YMRS- Young Mania Rating Scale; UPDRS- Unified Parkinson's Disease Rating Scale Motor Section (III); Time intervals correspond to time from the DBS operation. Within group changes were tested using the signed rank test and significant results are highlighted in bold (for p<0.05); while the between group comparisons were tested using the Wilcoxon rank sum test and corresponding p-values presented in the 3^rd^ row of each block.

**Table 5:** Comparison of Outcomes Over Time for all Bilateral STN and GPi DBS Cases.

Variable	Baseline (Median, Mean ± SD)	2-month			4-month			6-month			1-year		
		Median, Mean ± SD	Change	p-value	Median, Mean ± SD	Change	p-value	Median, Mean ± SD	Change	p-value	Median, Mean ± SD	Change	p-value
LED	700, 730±360				642, 715±267	−58.4, −30.9±247	0.547				500, 698±483	−58.4, −48.1±546	0.742
HAM-A	2.00, 3.44±4.88	4.50, 7.50±8.77	2.00, 3.63±5.66	0.172	7.00, 8.13±8.49	3.50, 4.25±6.09	0.117	6.50, 8.75±10.0	3.00, 4.88±7.62	0.125	6.00, 7.13±9.13	3.00, 3.25±5.20	0.125
HAM-D	2.00, 1.78±1.64	1.50, 4.00±5.63	0, 2.00±5.15	0.563	2.50, 4.50±5.42	1.00, 2.50±5.37	0.281	2.00, 4.50±4.78	1.50, 2.50±4.38	0.2031	3.00, 4.38±3.70	2.00, 2.38±2.97	0.078
BDI	4.00, 4.56±4.22				3.00, 2.78±2.49	−1.00, −1.78±4.74	0.359				4.00, 4.67±4.85	0, 0.11±4.17	0.969
AS	8.00, 8.11±5.35				8.00, 9.50±6.63	2.00, 1.75±6.56	0.469				11.0, 13.6±9.74	5.00, 5.88±9.45	0.141
YMRS	0, 1.56±3.68	3.00, 4.00±4.00	0.50, 2.25±3.15	0.125	3.00, 2.50±1.31	2.00, 0.75±3.85	0.438	2.50, 2.38±2.26	0.50, 0.63±2.26	0.5625	1.50, 1.50±1.41	0, −0.25±3.77	0.875
UPDRS Motor-Off	45.0, 47.1±17.3				30.0, 31.3±9.12	**−10.0, −15.8±13.6**	**0.004**				30.0, 28.0±8.76	**−15.0, −19.1±12.8**	**0.004**
UPDRS-Motor On	17.0, 22.6±11.2				25.0, 24.0±9.25	3.00, 1.44±12.1	0.375				26.0, 23.8±8.33	2.00, 1.22±9.35	0.758

LED- levodopa equivalent dose; HAM-A- Hamilton Anxiety Inventory; HAM-D- Hamilton Depression Inventory; BDI- Beck Depression Inventory; AS- Apathy Scale; YBOCS- Yale Brown Obsessive Compulsive Scale; YMRS- Young Mania Rating Scale; UPDRS- Unified Parkinson's Disease Rating Scale Motor Section (III); Time intervals correspond to time from the DBS operation. Significant changes are highlighted in bold (p<0.05; and for p<0.01).

**Table 6:** Spearman's Correlation Between Levodopa Equivalent Dose (LED) Change From Baseline to 1-year Compared to Mood and Behavior Changes.

Variable	Spearman's Correlation Coefficient	p-value
HAM-A	**0.518**	**0.007**
HAM-D	0.099	0.632
BDI	0.017	0.934
AS	−0.019	0.929
YBOCS	−0.134	0.522
YMRS	0.019	0.927
UPDRS Motor-Off	0.190	0.334
UPDRS-Motor On	0.045	0.820

## Discussion

The results of this study draw attention to the post-operative mood and behavioral changes that may emerge following unilateral and also staged bilateral STN and GPi DBS. The study was randomized by target and the results were stronger for the unilateral group because of the larger sample size. Importantly, when a staged unilateral approach was employed with regularly scheduled mood and apathy monitoring, and a non-aggressive medication reduction strategy (that resulted in an increased in dopaminergic dosage in the GPi group), few management issues emerged. The study revealed no statistically significant reduction of medications for either unilateral brain target, and we hypothesize, but cannot yet conclude that this impacted the occurrence of mood and apathy issues. There was a significant positive correlation between changes in dopaminergics and the anxiety and depression scores when re-checked at one year for both STN and GPi targets. The magnitude of the behavioral change was greatest in the anxiety domain. The dopaminergic dosage change was not correlated to mood and behavioral scores, and this finding could possibly be corroborated by a study looking specifically at bilateral DBS with larger dopaminergic dosage reductions. If dopaminergic dose reduction could be correlated with behavioral outcome, this would confirm the observations by Lhommee [12] and Thobois [11] who observed that following bilateral STN DBS when rapid and aggressive medication reduction was employed, there was an emergence of apathy, depressive symptoms, anxiety, and other behavioral features. Clinicians should be aware that though apathy and other behavioral issues may emerge with dopaminergic medication reduction, this trade-off may possibly be desirable to alleviate clinically disabling symptoms such as dyskinesia.

Though mood and apathy changes are features that most clinicians follow closely post-DBS, there were surprisingly few changes observed in these domains, and most of the issues resolved by the one year follow-up. Spread of electrical current outside the intended STN region is one potential mechanism proposed to result in mood changes [20]. Ventral STN stimulation in particular has been found to result in mood changes as shown in the original NIH COMPARE cohort, and in the current study patients were drawn directly from this dataset [13]. Two studies, COMPARE [13] and Anderson (2005) [21] revealed more adverse mood events in STN when compared to the GPi target. There has been speculation that the size differences and the surrounding fibers in the STN region may underpin some of the target specific differences [1], [6], [20]. In the VA Cooperative Study, GPi patients were happier, less angry, and less tired when compared to STN patients [22]. The current study did not examine at each interval the adverse event logs, visual analog scores, or the Profile of Mood States, which in previous studies, including our own, favored the GPi over the STN [13], [22].

The current study results differ slightly from the data derived from another recent subanalysis drawn from the NIH COMPARE cohort. Details of that subanalysis revealed that motor and mood scores at 6 months were unchanged, however apathy scores worsened. The difference when compared to our recent analysis likely resulted from the methodology employed. The former study used a longitudinal design-latent growth curve modeling, and also drew from a substantially larger sample size of 48 subjects. The time points collected were also slightly different. Apathy increased in both targets linearly from pre- to 6-months post-DBS by.66 points bi-monthly. The study concluded that “middle-aged adults (<65) had a steeper trajectory of apathy than older adults (≥65). Apathy trajectory was not related to motor severity, laterality of DBS, levodopa medication reduction, or motor changes after surgery.” [23]

The strength of the current study was the meticulous testing at multiple follow-up intervals post-DBS (2 weeks, 2 months, 4 months, 6 months and 1 year) and the use of validated scales. The weakness was the sample size, especially in the bilateral DBS cohort. Another weakness was not including the entire COMPARE cohort. However, it is important to appreciate that the lower numbers of bilateral GPi DBS cases was likely a byproduct of many GPi patients not requiring a second DBS lead. This finding was also reported in the original NIH COMPARE cohort [24]. Despite these flaws, the study was randomized, and outcomes were nearly identical in both targets (STN and GPi), and the data collectively demonstrated the relative safety of a unilateral staged DBS approach coupled with a post-operative strategy of non-aggressive medication reduction.

Post-DBS hypomania has been emerging as a potential concern in the field. The largest systematic study of this topic reported that 50% of patients (7/14) between 4 and 12 weeks post-STN DBS were documented to have hypomania. Shneider and colleagues reported the possibility that pre-DBS motor symptoms, global psychiatric symptom ratings, and illness severity all seemed related to the emergence of hypomania. [9] In another study, Chopra and colleagues observed that ventromedial DBS lead placement into a non-motor STN region, and the use of higher current densities were also possibly associated with hypomania, though sample sizes across the studies were small. [25] Some authors have suggested that switching to a more dorsolateral contact (i.e. moving stimulation into motor STN) may alleviate DBS induced hypomania. [26], [27] Finally, augmenting the medication regimen, [28] has been shown helpful in some cases. Though the mania scores increased in the current study, the small amount of change we observed has not to date been considered clinically meaningful. [29]

Though the STN and GPi subjects enrolled in this study experienced only mild changes in anxiety, the observations should not diminish the importance of emphasizing close post-operative monitoring. The finding of increased anxiety and behavioral side effects in either brain target in female subjects could be important only if it can be replicated with larger studies. It is likely that the close monitoring may have biased the results toward a more stable mood outcome. These findings support the overall findings of the NIH COMPARE DBS study, which revealed similar positive mood outcomes with STN or GPi DBS [13]. More frequent post-DBS follow-up visits likely resulted in enhanced optimization of medications, mood, and behavior, though this was not specifically tested in the current study. Another interesting aspect of this study was the lack of suicide or suicide attempts [30]. This finding may have been a byproduct of frequent clinical follow-up, small sample size, or by the non-aggressive medication reduction. Recently, Weintraub et. al. published a follow-up to the VA cooperative study which also revealed a lower than expected suicide and attempted suicide rate in bilateral STN and GPi DBS [31]. It is difficult in such a small study to draw any conclusions on suicide risk based on the selected management approach.

Overall, the results of this study must be interpreted with caution, though the target sites were randomized between STN and GPi making the findings potentially of greater significance. Since the original study's formal power analysis did not address the outcomes and time intervals for the current study, any findings of statistical significance could potentially change with increased sample frequency, longer follow-up time, or an increased number of subjects. Further hypothesis-driven studies will be critical to elucidate the non-motor changes following DBS, and a potential relationship to unilateral staged DBS (i.e. one side implanted and a second side added in another operative session), or to simultaneous bilateral implantations. This study did not address simultaneous bilateral DBS, impulse control disorder or dopamine dysregulation syndrome. The study was observational and was missing some baseline mood measures in a few patients, and this may have reduced the power of the analysis.

## Conclusions

Using a staged approach to STN or GPi DBS coupled with a strategy of non-aggressive medication reduction resulted in similar motor outcomes, and few adverse mood and apathy effects. The increased anxiety and mood issues were the most important finding, and though the sample size was small future studies should focus on female subjects. It is important to note that more dopaminergic medication was utilized in the GPi group. This increase in medication post-DBS could actually prove to be a long-term advantage over STN. Long term, the flexibility to adjust medications may be important for some patients, particularly those with dyskinesia. Clinicians should be aware of the potential increased risk of mood issues in patients when employing either DBS target. The approach of using staged STN or GPi DBS, offers an alternative to bilateral simultaneous STN DBS with aggressive medication reduction, however a direct comparison study, and especially a bilateral study is needed to better understand the differences between approaches.
